# IFN-λ: A new spotlight in innate immunity against influenza virus infection

**DOI:** 10.1007/s13238-017-0503-6

**Published:** 2018-01-13

**Authors:** Yeping Sun, Jingwen Jiang, Po Tien, Wenjun Liu, Jing Li

**Affiliations:** CAS Key Laboratory of Pathogenic Microbiology and Immunology, Institute of Microbiology, Chinese Academy of Sciences, Beijing, 100101 China

Influenza virus is a long-lasting and severe threat to human health. Seasonal flu epidemics, which are caused by the co-circulating influenza A viruses (IAVs) and influenza B viruses (IBVs), occur annually and lead to tens of millions of respiratory illnesses and up to half a million human deaths worldwide each year (Ginsberg et al., 2009). Influenza pandemics are more devastating. The 2009 swine-originated H1N1 virus, which caused the latest influenza pandemic, spread from Mexico and U.S. to virtually all countries throughout the world within only several months and was associated much higher mortality among children, young adults, and pregnant women than typical seasonal influenza viruses (Fineberg, 2014). The zoonotic avian influenza viruses, including H5N1, H5N6, H7N9 and H10N8, cause alarmingly high fatality rate in human cases, raising a public concern of pandemic influenza outbreak of avian origin (Poovorawan et al., 2013; Barr, 2017; Bui et al., 2017).

Innate immune system is an important barrier of defending against influenza virus infection. According to the traditional paradigm, after IAV gets across the mucus that covers the respiratory epithelium, it first invades and infects respiratory epithelial cells, from where it spreads to other non-immune and immune cells (e.g., macrophages and dendritic cells). In these cells, the virus can be sensed by the pattern recognition receptors (PRRs), triggering the production of type I interferons (IFNs) which induce the expression of hundreds of IFN-stimulated genes (ISGs) that block viral replication and further virus spread. Simultaneously, activation of PRRs also leads to production of pro-inflammatory cytokines (IL-6, IL-1β, IL-18, TNF, etc.) and chemokines. Pro-inflammatory cytokines induce topical and systemic inflammation, cause fever and anorexia, and direct the adaptive immune response against the virus. Chemokines, on the other hand, recruit innate immune cells (neutrophils, monocytes, and NK cells) which engulf and inactivate the virus, kill virally infected cells, and guide subsequent innate and adaptive immune responses that mediate ultimate viral clearance (Iwasaki and Pillai, 2014).

However, effective protection from influenza virus infection is provided by finely tuned antiviral immunity, while excessive innate immunity causes detrimental inflammation. Infection with influenza viruses is usually self-limited, though the severe cases, especially caused by highly virulent strains (e.g., the 2009 pandemic H1N1 virus, H5N1 and H7N9) are characterized by severe pulmonary disease and lethal acute respiratory distress syndrome (ARDS) (Bauer et al., 2006; Ramsey and Kumar, 2011; Ma et al., 2017). Influenza-induced ARDS, which involves the damage to the epithelial-endothelial barrier of the pulmonary alveolus, flute leakage to the alveolar lumen, and respiratory insufficiency, is associated not only with direct viral damage to epithelial-endothelial barrier, but also with inflammation mediated by components of the innate immune response. The cytokines produced in influenza virus infected epithelial and endothelial cells, and cytokines and reactive oxygen species produced by neutrophils and macrophages recruited to pulmonary alveolus all contribute to damage to the epithelial-endothelial barrier (Short et al., 2014).

Both the protective and pathological roles of innate immunity have been evidenced in human and experimental animals. On one hand, genetic deficiency in production of interferon or certain ISGs (e.g., MX and IFITM proteins) increases the vulnerability to IAV infection (Ciancanelli et al., 2015, 2016). On the other hand, genetic ablation of the pro-inflammatory cytokine IL-6 (Dienz et al., 2012), or chemical depletion of alveolar macrophages (Abboud et al., 2015), NK (Abboud et al., 2015) or neutrophils (Tate et al., 2009) in mice can exacerbate lung injury during IAV infection. These data support that both IFN response and inflammatory innate immune response are essential in protecting the host against IAV infections. However, gene expression analysis displays that early innate immunity signatures including pro-inflammatory cytokines (TNF, IL-1β, IL-6), chemokines (CCL2, CCL3, CCL4, CXCL1) and neutrophil infiltration are strongly associated with acute death of the mice infected with IAV (Brandes et al., 2013). In addition, deletion of pro-inflammatory cytokines IL-1, TNF and various chemokines decrease mortality in IAV-infected mice (Teijaro, 2015). Furthermore, hyper expression of pro-inflammatory cytokines and chemokines are linked to the high mortality in human cases infected with highly pathogenic influenza virus strains such as 1918 pandemic H1N1 virus and H5N1 virus (Loo and Gale, 2007; Peiris et al., 2009). These data suggest that exaggerate inflammation during lethal infection with IAV can have fatal consequences. Therefore, benign outcomes of influenza virus infection results from inducing sufficient IFN-mediated antiviral immunity while avoiding harmful inflammatory response.

Most current knowledge about innate immunity in influenza virus infection has come from IAV. Although IBV contributes a significant part to the burden arising from the worldwide epidemics of seasonal influenza, much less attention has been paid to IBV than IAV because IBV does not have a potential to cause a pandemic (Chai et al., 2017). IBV is characterized by strict host range limits, initiates local epidemics with a lower evolutionary rate and causes milder clinical syndrome than IAV (Jiang et al., 2016). However, little is known about the innate response against the IBV infection.

Recently, we investigated the global transcriptome of the human lung A549 cell line infected with IBV with RNA-seq. We identified 340 differentially expressed genes (DEGs) in these cells at 8 h after infection. Among them, we found that type III IFNs (including IFN-λ1, IFN-λ2, and IFN-λ3) were among the top highly up-regulated genes. However, type I IFNs (IFN-α/β) was not significantly up-regulated and not listed among the DEGs. Besides, various antiviral ISGs including almost all known antiviral effectors in the innate immune system, including OAS proteins, MX proteins, IFITM proteins, and viperin (Schneider et al., 2014), are also highly up-regulated.

**Figure 1:**
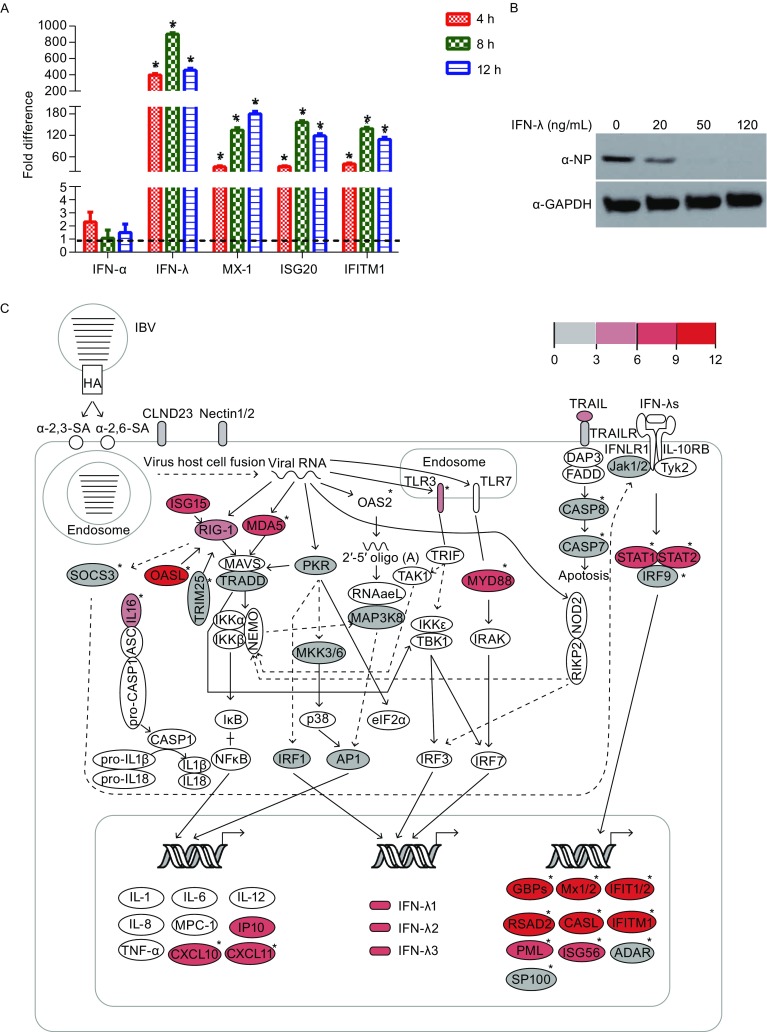
**The integrated signaling pathway for IBV infection in A549 cells**. (A) qRT-PCR analysis of the expression of selected genes in IBV infected A549 cells compared with uninfected controls. The fold difference was determined using the 2^−∆∆Ct^ method, and RNA levels were normalized to GAPDH. Error bars represent the standard deviation. (B) A549 cells were treated with recombinant human IFN-λ (R&D systems) at final concentration of 0, 20, 50 and 100 ng/mL, followed by infection with IBV for 12 h. Cell lysates were analyzed by western blot using indicated antibodies. (C) The pathways for IBV infection in A549 cells. The relative expression values are indicated by the color gradient. The full lines represent direct interactions, and the dashed lines represent indirect interactions. The uncolored ellipses represent genes that are not DEGs. ISGs are marked with an asterisk

To verify this, we performed a qPCR analysis to determine the expression level of IFNs genes, as well as three important antiviral ISGs: Mx1, ISG20 and IFITM1 at 4, 8, and 12 h post infection. Consistent with the RNA-seq data analysis, IFN-λ expression was significantly increased at all the time points. The Mx1, ISG20 and IFITM1 also displayed markedly altered mRNA levels during IBV infection. In contrast, the expression of IFN-α was not altered at all points (Fig. 1A). Since the function of both IFN-λ and IFN-α is induction of ISG expression *via* the same intracellular signaling pathways, we speculate that it is IFN-λ rather than IFN-α that dominates the production of ISGs in IBV-infected A549 cells. Indeed, the IBV NP protein levels were obviously decreased in IFN-λ stimulated A549 cells, indicating the potent antiviral activity during IBV replication in A549 cell (Fig. 1B).

**Figure 2:**
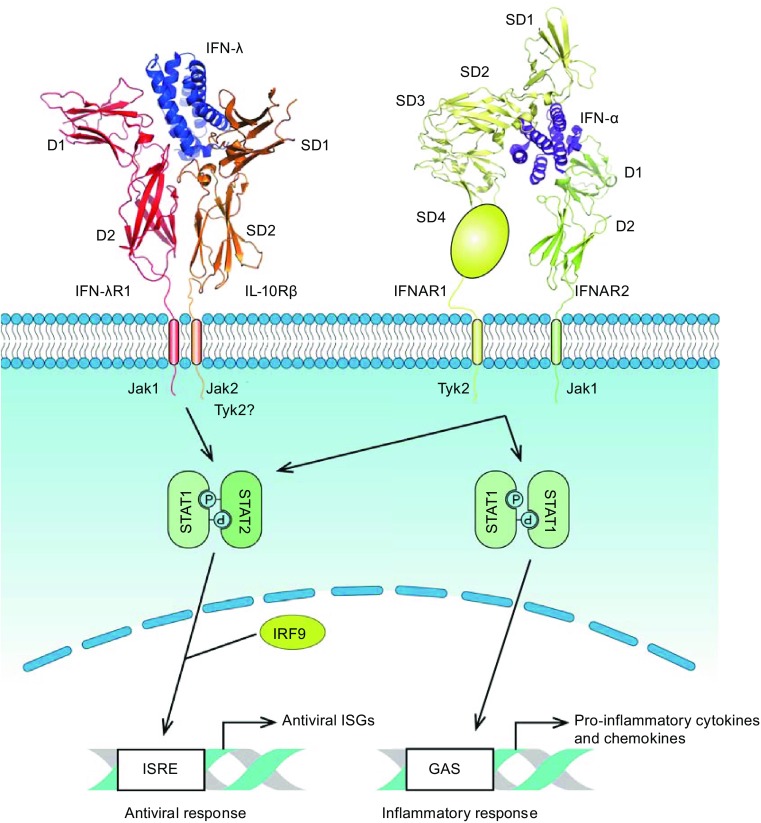
**Different gene expression pattern triggered by type I and type III IFNs**. Type III IFNs (IFN-λ3, PDB ID: 5T5W) binding to their heterodimeric receptor composed of IFNλR1 and IL-10Rβ triggers the formation of heterdimeric complex of STAT1 and STAT2 which translocates to the nucleus where it forms ternary complex with IRF9 and form IFN-stimulated gene factor 3 (IGF3). IGF3 binds to IFN-stimulated response elements (ISREs) and induces the expression of antiviral ISGs. Engagement of type I IFNs (IFN-α2, PDB ID: 3SE3) with their receptor, the heterodimer composed of IFNAR1 and IFNAR2, can also activate the STAT1 homodimer which binds to gamma-activated sequence (GAS) and induces the expression of pro-inflammatory cytokines and chemokines

Based on the above results and KEGG pathways assay, we built the intracellular signaling pathway related to IBV infection in A549 cells (Fig. 1C). IBV infection begins with viral HA binding to α-2,3 or α-2,6 sialic acid on the host cell surface. After binding to the receptor, the virus enters the endosome, where the acidic environment triggers virus-host cell membrane fusion, after which the viral RNA is released into the cytoplasm. In the cytoplasm, the virus-derived RNA is recognized by multiple canonical pattern recognition receptors (PRRs), including the RIG-I-like receptors (RLRs, RIG-I, and MD5), the Toll-like receptors (TLRs, TLR-3, and 7), the NOD-like receptors (NLR and NOD1/2), and the PKR, and activates their respective signaling pathways. Activation of these pathways leads to the expression of IFN-λs, which then interact with their receptor, the heterogeneous dimer IFNLR1/IL-10R, and activate the expression of ISGs through the JAK/STAT pathway.

Interferons (IFNs) are classified into three categories (type I IFNs, type II IFNs, and type III IFNs). All IFNs signal through JAK/STAT pathway which leads to transcription of ISGs, but their production cells and receptor specificity are different (Chow and Gale, 2015). Type I IFNs include IFN-α, β, ε, κ, and ω. They can be produced by almost all cell types and their receptor is the widely-expressed heterodimer composed of IFNR1 and IFNR2. Type II IFN is INF-γ, which is produced by immune cells and forms dimmer to act on the receptor complex consisting of two IFNR1 and two IFNR2. Type III IFNs include IFN-λ1 (IL-29), IFN-λ2 (IL-28A), IFN-λ3 (IL-28B), and IFN-λ4. Their receptor is the heterodimeric complex composed of IFN-λR1 and IL-10Rβ. IL-10Rβ is broadly distributed, but the expression of IFN-λR1 is mainly restricted to epithelial cells (Broggi et al., 2017).

Although discovered later than type I and type II IFNs, type III IFNs in innate immunity are attracting more and more attention. The potential importance of type III IFNs has been suggested by life-threatening influenza developed in genetically deficiency that leads to impaired production of both type I and type III IFN production in a 9-year-old child (Ciancanelli et al., 2015). IFNLR is expressed by human hepatocytes, and IFNL polymorphisms are associated with ameliorated outcome from HCV and HBV infection (Bibert et al., 2013; Galmozzi et al., 2014). Clinical trials have displayed that the pegylated IFN-λ can reduce the level of HCV and HBV in patients infected with chronic HCV and HBV, respectively (Zeuzem et al., 2011; Phillips et al., 2017). IFN-λs are also highly expressed in gastrointestinal tracts, and can cure chronic murine norovirus infection without the presence of adaptive immunity (Nice et al., 2015).

Most recently, several important breakthroughs have increased the brightness of IFN-λs as a new spotlight in innate immunity. One of them is the determination of the crystal structure of an engineered high-affinity IFN-λ3 (H11) in complex with its two receptors, IFN-λR1 and IL-10R. In this structure, the IFN-λ3 helical bundle binds to the gorge formed by the two domains at the distal ends of its two receptors to the cellular membrane. In contrast, the two receptors bind to the opposite sides of the helical bundles of type I IFNs (Mendoza et al., 2017).

Another breakthrough is the definition of the differential roles of IFN-λs in contrast to type I IFNs in IAV infection using Ifnlr1^−/−^, Ifnαr1^−/−^ and Ifnlr1^−/−^ Ifnαr1^−/−^ mice (Galani et al., 2017). This study shows that in mice infected with IAV, IFN-λs emerge earlier than type I IFNs and show potent protective function during the early phase of the infection, when type I IFNs are still scarce. Furthermore, IFN-λs treatment induces expression of antiviral genes but not pro-inflammatory cytokines in neutrophils, while type I IFNs induce the expression of both types of genes and are involved in causing inflammation and immunopathology.

The different functions between IFN-λs and type I IFNs can be explained by the different STAT complex they activated. When engaged with their receptor complexes, both IFN-λs and type I IFNs activate JAK-family kinases, which phosphorylate STAT1 and STAT2. The phosphorylated STAT1 and STAT2 dimerize and translocate to the nucleus where they associate with IRF9 to form IFN-stimulated gene factor 3 (ISGF3), bind to IFN-stimulated response elements (ISREs) and induces the expression of antiviral ISGs. However, engagement of type I IFNs with their receptor can also activate the STAT1 homodimer which binds to gamma-activated sequence (GAS) and induces the expression of pro-inflammatory cytokines and chemokines (Ivashkiv and Donlin, 2014). Thus, type I IFNs have the potential of inducing inflammation in addition to antiviral function, while IFN-λs promote the production of antiviral ISGs without the function of inducing inflammation (Fig. 2).

In summary, IFN-λs are more advantageous than type I IFNs in defending against influenza virus for several reasons: (1) IFN-λs are produced earlier and in larger amount during influenza virus infection; (2) unlike type I IFNs, IFN-λs are produced and act specifically in respiratory tract, so they do not induce systematic side effects; (3) type III IFNs have potent antiviral activity without mediating inflammation. Therefore, IFN-λs share the therapeutic benefits but eschews many side effects associated with the clinical use of IFN-α/β (Lazear et al., 2015). In the future, scientists should explore the possibility that whether IFN-λs are more ideal therapeutic reagents for dealing with respiratory tract infection by viruses like IBV or not.
